# An investigation of fentanyl and methamphetamine use among first-time arrestees from 25 county jails across the U.S. in 2023

**DOI:** 10.1186/s13722-025-00588-5

**Published:** 2025-07-24

**Authors:** Joseph E. Schumacher, Abdullah Ahsan, Amber H. Simpler, Adam P. Natoli, Bradley J. Cain, Peter S. Chindavong, Aren Yarcan

**Affiliations:** 1 NaphCare Charitable Foundation, Inc., 2101 Old Columbiana Road, Suite 100, Birmingham, AL 35216 USA; 2 Natoli Services, LLC, 1315 10th St., PO Box 1192, Huntsville, TX 77342 USA; 3 Alabama College of Osteopathic Medicine, 445 Health Sciences Boulevard, Dothan, AL 36303 USA

**Keywords:** Opioid, Fentanyl, Methamphetamine, Jail, Arrestees, Substance use

## Abstract

Widespread use of fentanyl in combination with methamphetamine in carceral settings presents unique health risks and public health challenges. To contribute to continued efforts to understand drug use among first-time arrestees, this study characterizes the nature and extent of urine drug screenings (UDS) positive for fentanyl and/or methamphetamine among first-time arrestees receiving healthcare in 25 jails across the U.S. This study used the same data source, data extraction, sample selection, and UDS variables as those reported by Schumacher et al. (2025) and a similar data analytic strategy which included 81,842 arrestees with a UDS or 28.8% of total arrestees (283,884). Among first-time arrestees with complete results (43,553), 32,561 or 74.8% of arrestees tested positive for any drug and among those, 14,426 (44.3%) were positive for fentanyl and/or methamphetamine. Of those, 59.8% and 11.5% were only positive for methamphetamine or fentanyl, respectively, while 28.7% were positive for both. Demographically, individuals testing positive for both fentanyl and methamphetamine were predominantly white young adults (aged 20–39), with similar co-occurrence patterns in males and females. Fentanyl alone was more common in southern and midwestern jails and mega-sized jails, methamphetamine was more common in medium-large and southern jails, and their co-occurrence was most common in western and large jails. Approximately 97.5% of first-time arrestees tested positive for two or more drugs, with individuals testing positive for five or more drugs significantly more likely to test positive for both fentanyl and methamphetamine. This study highlights the significant prevalence of methamphetamine and/or fentanyl use among first-time arrestees, underscoring the urgent need for targeted interventions, improved in-jail substance use treatment, and post-release support to mitigate overdose risks and enhance public health outcomes.

## Introduction

The ongoing opioid epidemic in the U.S. has progressed through distinct waves: the first (2000–2016) saw rising overdose deaths from prescription opioids, the second (2007) involved a shift to heroin, the third (2013) introduced synthetic opioids such as fentanyl, and the current fourth wave is marked by polysubstance use, particularly fentanyl combined with stimulants [4, 5, 9]. Opioid-related overdose deaths surged from 2010 to 2021, disproportionately affecting older African Americans and individuals in the Western U.S. [9]. Methamphetamine-related deaths have also risen sharply, with a 50-fold increase from 1999 to 2021 [14]. Moreover, in 2021, 61.2% of these cases also involved heroin or fentanyl. The spread of illicit fentanyl, particularly post-COVID-19, has driven increased stimulant-related overdoses, often through unknown consumption [5, 6, 8, 17]. These trends reflect overlapping drug epidemics, each shaped by distinct demographic and geographic factors [15].

The above statistics are alarming, and the deleterious effects of these overlapping drug epidemics are particularly concerning in correctional settings. Incarcerated individuals have been found to be twice as likely to evidence higher fentanyl and/or methamphetamine levels than the general population, with higher odds of fentanyl overdose in the incarcerated population as well [13]. In fact, fentanyl-related overdoses have been increasing in correctional settings over the last decade [16]. In Jefferson County, Kentucky, for instance, fentanyl has become a leading cause of overdose deaths among recently incarcerated individuals, with methamphetamine involvement exceeding that of non-incarcerated individuals [13]. Justice-involved individuals also show high rates of polysubstance use, with some jails evidencing 13.2% of arrestees involving concurrent opioid and stimulant use [2] while others are seeing upwards of 33% of arrestees testing positive for multiple substances—notably, 97% for methamphetamine and 21% for opiates [21]. To help shed light onto these trends as they occur among first-time arrestees, a recent multi-jail study examined the results of urine drug screens (UDS) from 43,533 first-time arrestees booked through jails across the U.S. in 2023. Schumacher and colleagues [22] found a 74.8% positivity rate for any drug among tested arrestees, with cannabis (69.0%), stimulants (29.6%), opioids (26.9%), and sedatives (12.4%) being the most common. Nearly half (48.9%) tested positive for multiple substances, most commonly cannabis and stimulants (53.0%), followed by opioids and stimulants (25.1%).

With the above literature as context, it’s clear that jails are critical settings for screening, assessment, and treatment interventions targeting drug use, particularly for first-time arrestees. The current wave of polysubstance use, particularly fentanyl combined with stimulants, is sweeping across the U.S. with devastating effects in correctional settings. Yet, there is more to understand about the nature and extent of this Fourth Wave of the opioid crisis. Investigating fentanyl and methamphetamine use among first-time jail arrestees in a healthcare context can provide important insights and inform more effective strategies for substance use screening, clinical assessment, and treatment [13].

### Current study

As a continuation of a line of inquiry investigating and documenting drug use among first-time arrestees from a jail healthcare perspective, the purpose of the present study is to characterize the nature and extent of UDS results positive for fentanyl and/or methamphetamine among contemporary first-time arrestees receiving healthcare in 25 jails across the U.S. by:

1. Reporting the UDS positivity rate of fentanyl, methamphetamine, and combined fentanyl and methamphetamine among first-time jail arrestees undergoing healthcare screening and UDS testing;
2. Comparing multiple fentanyl and methamphetamine UDS positivity groups and subgroups across jail characteristics and arrestee demographics; and
3. Exploring the frequency and rank order of fentanyl and methamphetamine UDS positivity when combined with other drugs.

## Method

This study used the same data source, data extraction, sample selection, and UDS variables as those reported by Schumacher et al. [22], and a similar data analytic strategy. The following subsections summarize these procedures and detail adaptations or departures from the previous methodology.

### Data source

This study included county government-operated jails across the U.S. served by a correctional healthcare provider of medical, mental health, and pharmacy services (*NaphCare, Inc.*). Individuals accused of criminal offenses or convicted of minor/misdemeanor crimes (*arrestees*) who underwent screening while held in custody were included in this study. Data related to the screening, diagnosis, and management of health conditions, including drug use and UDS results, among these arrestees were collected as part of routine care and documented in a proprietary electronic health record (EHR) software system (*TechCare 5.0*). The researchers obtained these clinical data on arrestees in a de-identified manner under a confidential data-sharing agreement. This study was deemed exempt from institutional review board as the secondary data analysis of archival, anonymized data did not meet the definition of human subjects research (per Salus IRB, Case ID #23102-01).

Out of the 30 secure facilities where the healthcare company was contracted to provide healthcare services in 2023, 25 jails met the following study inclusion criteria: (1) facilities with an active contract with the correctional healthcare provider throughout 2023; (2) facilities where arrestees with a self-reported history of drug use or suspicion of withdrawal risk consented to undergo the UDS in 2023; and (3) facilities that recorded UDS results from 2023.

Distribution of the 25 study jails spanned the Western, Midwestern, and Southern regions defined by the U.S. Census Bureau, but excluded the Northeast, limiting generalizability across all four major U.S. regions [22]. Jail size was defined by contractual bed capacity and categorized into three groups as follows: Medium-Large (250–499), Large (500–999), and Mega (≥ 1,000). Of the 25 jails included, 12 were located in the West (48%), five in the Midwest (20%), and eight in the South (32%). Jail size distribution included eight Medium-Large (32%), 10 Large (40%), and seven Mega (28%) jails.

Compared to national jail demographics [25], the Schumacher et al. [22] sample had a higher proportion of females (36.6% vs. ~ 10–15%), a similar overrepresentation of Black individuals (34.3% vs. ~ 33%), and a comparable age distribution, with most arrestees aged 20–39 (60.3%). In terms of drug screening policies, the participating jails used a systematic UDS protocol for arrestees at risk of withdrawal, a more structured practice than the national average, where only approximately 25% of U.S. jails conduct routine drug testing [22, 24].

### Data extraction

The de-identified jail characteristics, arrestee demographics, and UDS results among arrestees across the 25 jails between 1 st January 2023 and 31 st December 2023 were extracted from the EHR system using Structured Query Language (SQL) scripts/queries and exported in Excel files. After creating two separate pandas DataFrames—one for demographics and jail characteristics and one for UDS results—descriptive statistics were computed using Python and its libraries to generate appropriate frequencies, tables, and figures.

### Study population

All arrestees across the 25 jails underwent screening assessments for acute and chronic health conditions at the time of booking and were triaged for additional assessments as necessary. However, only those arrestees who self-reported or were suspected to be using or chemically dependent on substances such as alcohol, benzodiazepines, or opioids and potentially at risk of withdrawal were approached for a UDS within 48 h of booking. As described elsewhere (see [22]), withdrawal risk was determined by healthcare staff through observations, review of arrestees’ self-reported substance use, history of prescription medicine misuse or medically assisted drug detoxification, symptoms of drug withdrawal, and/or drug-related hospitalizations.

Since UDS administration was limited to arrestees self-reporting and/or otherwise believed to be at risk of withdrawal or needing withdrawal management, the true frequency of drug use cannot be determined. Instead, these data are best understood as reflecting drug use frequency and patterns as known to correctional and healthcare staff, which constitute the information guiding interventions and policy decisions in these naturalistic settings. The collection, observation, analysis, and EHR documentation of UDS samples were conducted by on-site healthcare professionals.

### Urine drug screen (UDS)

Urine samples were screened with the Quick Test Cup™ Multi-Drug Urine Cup (MD-U261) and the Rapid Test Strip, Urine, for fentanyl (FYL-U11) manufactured by 12 Panel NOW™. MD-U261 test results were negative or presumed positive for the following drugs: opioid (OPI), methamphetamine (MET), amphetamine (AMP), benzodiazepine (BZO), cocaine (COC), methadone (MTD), oxycodone (OXY), marijuana (THC), barbiturates (BAR), buprenorphine (BUP), 3,4-Methyl​enedioxy​methamphetamine or “Ecstasy” (MDMA), and phencyclidine (PCP).

### FEN/MET positivity groups and subgroups and sample sizes

**Fig. 1:**
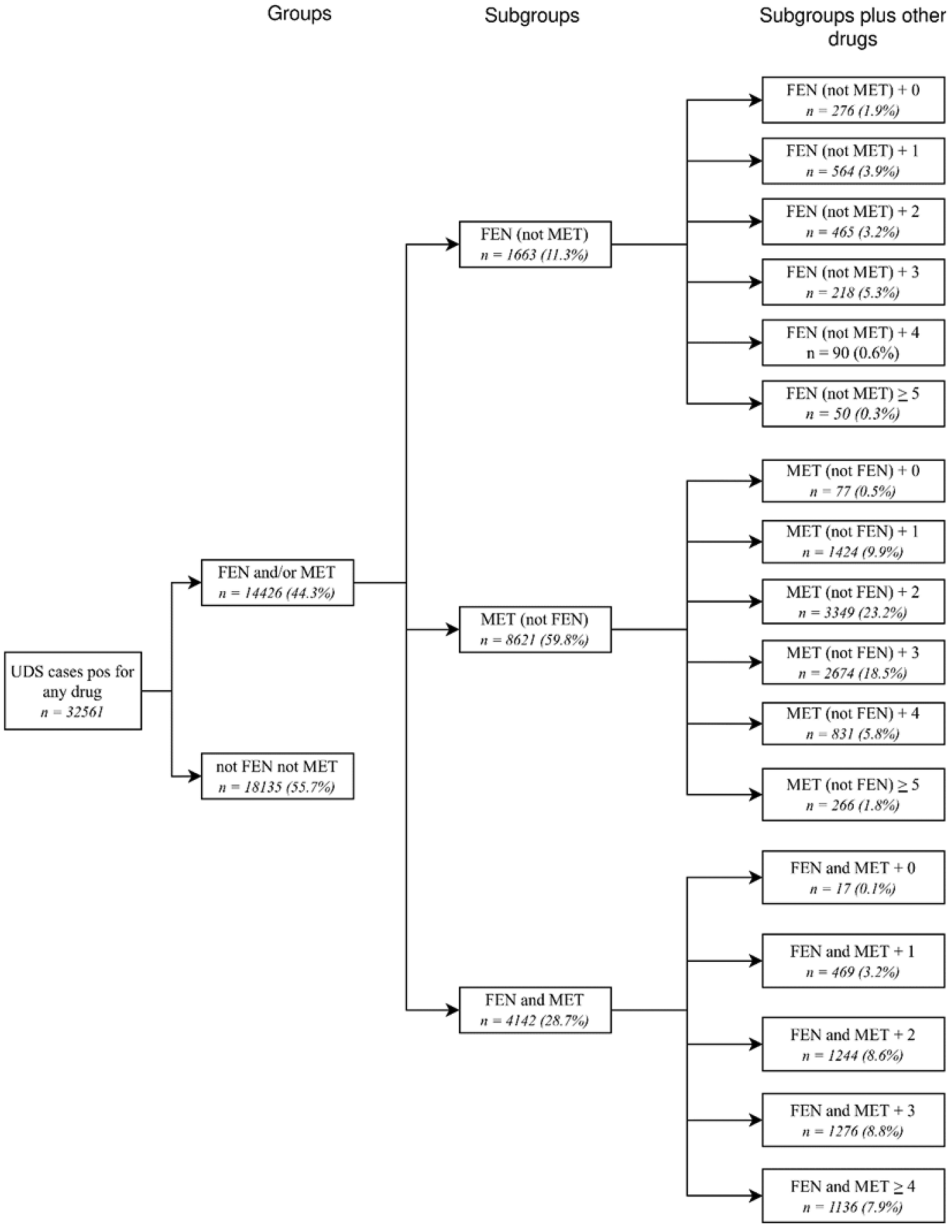
Breakdown and sample sizes for FEN/MET groups, subgroups, and subgroups plus other drugs. Variable definitions are found in the Methods section

Figure 1 provides a breakdown of fentanyl and methamphetamine groups, subgroups, and subgroups plus other drugs and their sample sizes from the total sample of UDS cases positive for any drug (*N* = 32,561). UDS cases positive for any drug were divided into two groups: (1) UDS for fentanyl and/or methamphetamine were positive (FEN and/or MET) or (2) neither fentanyl nor methamphetamine UDS were positive (not FEN not MET). FEN and/or MET was further separated into three subgroups: UDS for fentanyl was positive, but negative for methamphetamine—FEN (not MET); UDS for methamphetamine was positive, but negative for fentanyl—MET (not FEN); and UDS for both fentanyl and methamphetamine were positive—FEN and MET. Lastly, each subgroup (total of 17) was categorized based on the number of drugs other than FEN or MET used.

### Sample selection

Building on prior work focused on first-time arrestees (e.g., [22]), the current sample was limited to individuals who, according to available records, were arrested and received a urine drug screen (UDS) for the first time in 2023. Of the 283,884 arrestees identified that year, 81,842 (28.8%) underwent UDS. From these, we included only those with complete UDS results (77,074,27.1%) and no prior arrest history before 2023 (50,524; 17.8%). Arrestees were excluded if they did not undergo UDS, had missing results for any of the 13 substances tested, or had a prior arrest. For individuals with multiple arrests or UDS in 2023, only the first UDS was retained. This yielded a final sample of 43,533 first-time arrestees with a complete UDS, of whom 74.8% (n = 32,561) tested positive for any drug. Among these, 44.3% (n = 14,426) were positive for fentanyl (FEN), methamphetamine (MET), or both, while 55.7% (n = 18,135) were not. As shown in Fig. 1, this study focused on the subsample of arrestees with UDS positive for FEN and/or MET.

### Statistical analyses

After determining (sub)sample arrestee demographics (see Table 1), frequencies and percentages were calculated for FEN and/or MET, FEN (not MET), MET (not FEN), and FEN and MET subgroups and partitioned by jail characteristics and arrestee demographics (see Table 2). To gain insight into patterns of the UDS results, a series of Pearson’s chi-square tests of independence were performed to statistically analyze associations between UDS results and jail characteristics and arrestee demographics. Significant chi-square test results were probed by examining standardized residuals to clarify the nature and significance of within-group differences in proportions. Standardized residuals greater than zero were interpreted as indications of higher prevalence of certain drug combinations than expected for a specific subgroup (i.e., a greater likelihood than expected under the null), and those less than zero signifying a frequency smaller than expected under the null. Absolute standardized residuals equal to or greater than 1.96 were deemed significantly different from zero (i.e., at p < 0.05).

**Table 1:** Frequencies and percentages of urine drug screen (UDS) cases positive for any drug and fentanyl/methamphetamine (FEN/MET) groups by jail characteristics and arrestee demographics

Variable	UDS positive for any drug^a^ *N* = 32,561		FEN/MET groups^b^			
			FEN and/or MET *n* = 14,426 44.3%		Not FEN not MET *n* = 18,135 55.7%	
	*n*	%	*n*	%	*n*	%
Jail characteristics						
Jail location						
West	13176	40.5	7490	51.9	5686	31.4
South	10241	31.5	3643	25.3	6598	36.4
Midwest	9144	28.1	3293	22.8	5851	32.3
Jail size						
Medium–large	4322	13.3	2550	17.7	1772	9.8
Large	15027	46.2	6679	46.3	8348	46.0
Mega	13212	40.6	5197	36.0	8015	44.2
Arrestee demographics						
Sex						
Male	20645	63.4	9402	65.2	11243	62.0
Female	11916	36.6	5024	34.8	6892	38.0
Race						
White	17745	54.5	10360	71.8	7385	40.7
Black	10917	33.5	2374	16.5	8543	47.1
Hispanic	127	0.4	58	0.4	69	0.4
Asian	372	1.1	227	1.6	145	0.8
Other	55	0.2	43	0.3	12	0.1
Unknown	3345	10.3	1364	9.5	1981	10.9
Age groups						
Adolescent (age 15–19)	1399	4.3	198	1.4	1201	6.6
Young adult (age 20–39)	19946	61.3	8548	59.3	11398	62.9
Adult (age 40–59)	9905	30.4	5214	36.1	4691	25.9
Senior (age 60 or older)	1311	4.0	466	3.2	845	4.7

Variable definitions for FEN/MET groups are found in the Method section. Geographical location of jails was coded according to the census regions of the U.S. Census Bureau 2020) (Northeast not represented). Jail size categories were based on contracted bed capacity of a given facility: Medium-Large (250–499), Large (500–999), and Mega (≥ 1,000). “Other” race categories included individuals identified as Indigenous, Hawaiian, and South Asian. Unknown race was not assessed or documented

^a^Positive UDS percentages were calculated by dividing (and multiplying by 100) the number of positive UDS cases for each jail characteristic and arrestee demographic variable by the total number of positive UDS cases

^b^Percentages for FEN/MET groups were calculated by dividing (and multiplying by 100) the number of UDS results for FEN and/or MET and not FEN not MET for each jail characteristic and arrestee demographic variable by the total number of positive UDS cases

**Table 2:** Frequencies and percentages of urine drug screen (UDS) cases positive fentanyl and/or methamphetamine (FEN and/or MET) and three FEN and/or MET subgroups by jail characteristics and arrestee demographics

Variable	FEN and/or MET *n* = 14,426	FEN and/or MET Subgroups^a^					
		FEN (not MET) *n* = 1663 11.5%		MET (not FEN) *n* = 8621 59.8%		FEN and MET *n* = 4142 28.7%	
	*n*	*n*	%	*n*	%	*n*	%
Jail characteristics							
Geographic location							
West	7490	412	5.5	4380	58.5	2698	36.0
South	3643	550	15.1	2300	63.1	793	21.8
Midwest	3293	701	21.3	1941	58.9	651	19.8
Jail size							
Medium–large	2550	155	6.1	1607	63.0	788	30.9
Large	6679	689	10.3	3856	57.7	2134	32.0
Mega	5197	819	15.8	3158	60.8	1220	23.5
Arrestee demographics							
Sex							
Male	9402	1104	11.7	5630	59.9	2668	28.4
Female	5024	559	11.1	2991	59.5	1474	29.3
Race							
White	10360	1010	9.7	6027	58.2	3323	32.1
Black	2374	458	19.3	1556	65.5	360	15.2
Hispanic	58	4	6.9	39	67.2	15	25.9
Asian	227	7	3.1	170	74.9	50	22.0
Other	43	3	7.0	30	69.8	10	23.3
Unknown	1364	181	13.3	799	58.6	384	28.2
Age group							
Adolescent (age 15–19)	198	56	28.3	93	47.0	49	24.7
Young Adult (age 20–39)	8548	1081	12.6	4612	54.0	2855	33.4
Adult (age 40–59)	5214	446	8.6	3597	69.0	1171	22.5
Senior (age 60 or older)	466	80	17.2	319	68.5	67	14.4

Variable definitions for FEN and/or MET subgroups are found in the Method section. Geographical location of jails was coded according to the census regions of the U.S. Census Bureau 2020) (Northeast not represented). Jail size categories were based on contracted bed capacity of a given facility: Medium-Large (250–499), Large (500–999), and Mega (≥ 1,000). Race Others include Indigenous, Hawaiian, and South Asian. Unknown race was not assessed or documented

^a^Percentages for FEN and/or MET subgroups were calculated by dividing (and multiplying by 100) the number of UDS results positive for a given pattern by the total number of positive FEN and/or MET cases for each jail characteristic and arrestee demographic variable

## Results

### Sample descriptive statistics

Table 1 presents jail characteristics and arrestee demographics for 32,561 first-time arrestees with a UDS positive for any drug and divided into those testing positive for FEN and/or MET and not FEN not MET groups. The overall sample was fairly equally distributed across the three geographical regions and larger proportions of arrestees were housed in Large and Mega jails versus Medium-Large jail sizes, as would be expected. The overall sample was primarily comprised of young adult males identified racially as White (see [22], for an in-depth analysis of this larger sample).

Table 1 also shows 44.3% of the overall sample (*n* = 14,426) tested positive for FEN and/or MET and 18,135 (55.7%) did not. Table 2 shows that among those positive for FEN and/or MET, 1,663 (11.5%) tested positive for FEN (not MET), 8,621 (59.8%) positive for MET (not FEN), and 4,142 (28.7%) positive for FEN and MET.

### Proportional differences in positive UDS results for fentanyl/methamphetamine (FEN/MET) groups and subgroups by jail characteristics and arrestee demographics

**Fig. 2:**
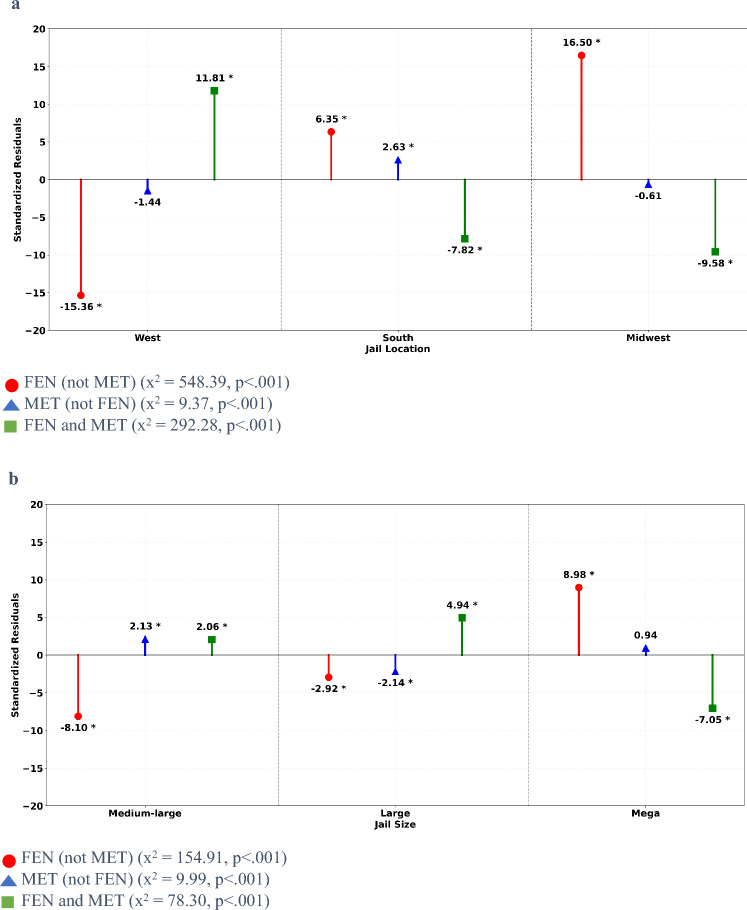
**a** Standardized residuals for jail location by fentanyl (FEN) and methamphetamine (MET) co-occurrence patterns. Chi-square results reflect tests of association between for a given co-occurrence pattern and specified jail characteristics. *Difference in proportions significant at p < 0.05 based on standardized residual. **b** Standardized residuals for jail size by fentanyl (FEN) and methamphetamine (MET) co-occurrence patterns. Chi-square results reflect tests of association between for a given co-occurrence pattern and specified jail characteristics. *Difference in proportions significant at p < 0.05 based on standardized residual

Jail Characteristics. Examination of the overall study sample revealed a significant association between jail location and UDS results positive for FEN and/or MET, χ^2^_(2)_ = 1411.06, *p* < 0.001. Standardized residuals illustrated the proportion of UDS results positive for FEN and/or MET was significantly larger than expected in Western jails (*z* = 21.63) and significantly smaller than expected in both Southern (*z* = −13.28) and Midwestern (*z* = −11.91) jails. Significant associations between jail location and UDS results were observed across the three FEN and/or MET subgroups: FEN (not MET), MET (not FEN), and FEN and MET, χ^2^_(4)_ = 850.04, *p* < 0.001. Standardized residuals depicted in Fig. 2a show a significantly greater than expected proportion of UDS results in Western jails were positive for FEN and MET, whereas the proportion of UDS results in Southern and Midwest jails positive for FEN and MET were significantly smaller than expected. The proportion of FEN (not MET) UDS results was significantly smaller than expected in Western jails and larger than expected in Southern and Midwestern jails, whereas the proportion of MET (not FEN) UDS results in Southern jails was significantly larger than expected and not significantly different than expected in either Western or Midwestern jails.

Jail size was also found to be significantly related to UDS results positive for FEN and/or MET in the overall study sample, χ^2^_(2)_ = 510.61, *p* < 0.001, with significantly greater frequency in Medium-Large jails (*z* = 14.51), significantly smaller frequency in Mega jails (*z* = −8.58), and approximately at a level expected in Large jails (*z* = 0.26). Likewise, jail size was significantly associated with UDS results across the three FEN and/or MET subgroups, χ^2^_(4)_ = 243.22, *p* < 0.001 (see Fig. 2b). The proportion of UDS results positive for FEN (not MET) were significantly less than expected in Medium-Large and Large jails, but significantly larger than expected in Mega jails. The opposite was observed for UDS results positive for both FEN and MET. Although the proportion of MET (not FEN) UDS results in Mega jails were as expected under the null, significantly more MET (not FEN) results were seen in Medium-Large jails and significantly fewer than expected were seen in Large jails.

**Fig. 3:**
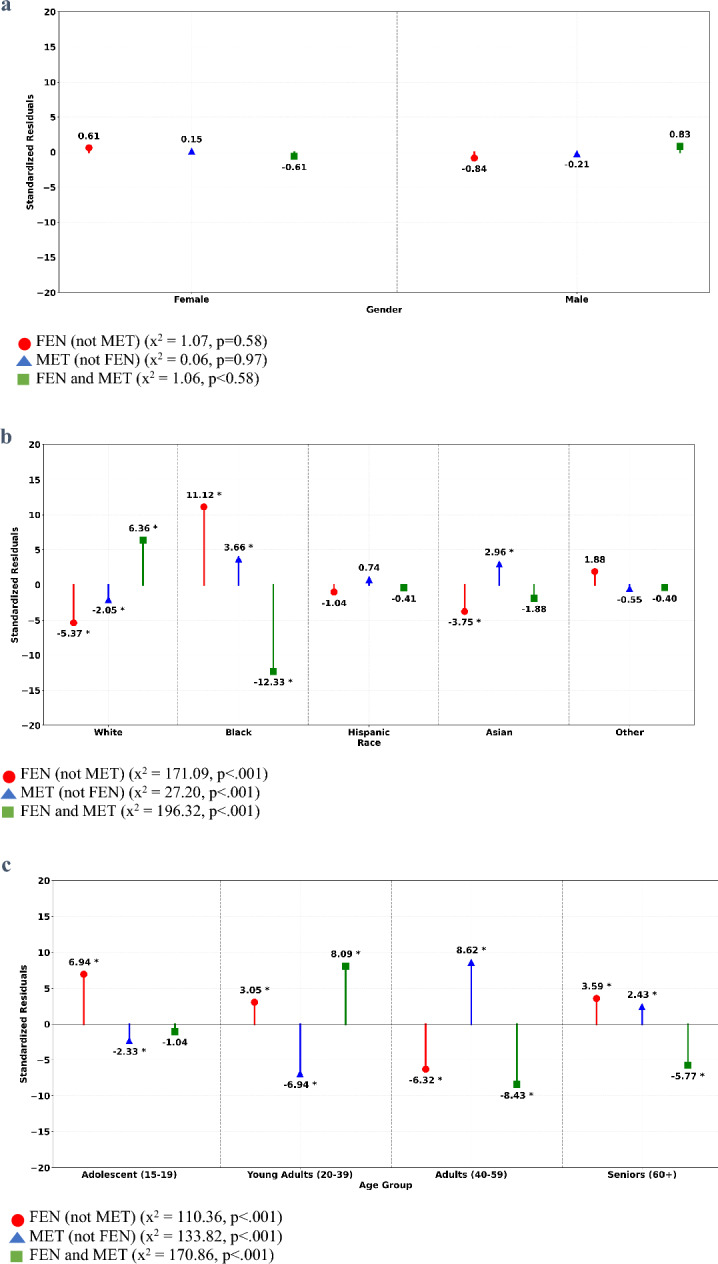
**a** Standardized residuals for sex by fentanyl (FEN) and methamphetamine (MET) co-occurrence patterns. Chi-square results reflect tests of association between for a given co-occurrence pattern and specified jail characteristics. *Difference in proportions significant at p < 0.05 based on standardized residual. **b** Standardized residuals for race by fentanyl (FEN) and methamphetamine (MET) co-occurrence patterns. Chi-square results reflect tests of association between for a given co-occurrence pattern and specified jail characteristics. *Difference in proportions significant at p < 0.05 based on standardized residual. **c** Standardized residuals for age by fentanyl (FEN) and methamphetamine (MET) co-occurrence patterns. Chi-square results reflect tests of association between for a given co-occurrence pattern and specified jail characteristics. *Difference in proportions significant at p < 0.05 based on standardized residual

Arrestee Demographics. The proportion of overall UDS results positive for FEN and/or MET among males was 0.455 and 0.422 among females. This difference in proportions was significant, χ^2^_(1)_ = 34.97, *p* < 0.001. Based on odds ratios, the odds of a female arrestee testing positive for FEN and/or MET was approximately 14.7% higher compared to a male arrestee (OR = 1.15; 95% CI [1.10, 1.20]). As Fig. 3a shows, there were no significant differences in proportions between male and female arrestees across the three FEN and/or MET subgroups, χ^2^_(2)_ = 2.20, *p* = 0.333.

There was a significant association between arrestee race and the proportion of UDS results positive for FEN and/or MET in the overall sample, χ^2^_(5)_ = 3761.38,* p* < 0.001. Generally, significantly more than expected FEN and/or MET positive UDS results were observed among White (*z* = 28.17), Asian (*z* = 4.84), and Other Race (*z* = 3.77) arrestees whereas the opposite was seen for arrestees who were Black (*z* = −35.41) or for whom race was Unknown (*z* = −3.06); no significant association was observed for Hispanic arrestees (*z* = 0.23).

Due to an expected frequency less than five for the Unknown Race group in the FEN (not MET) condition (expected frequency = 4.96), the Unknown Race group was removed prior to examining proportional differences between races for the three FEN and/or MET subgroups. Significant proportional differences were found across the remaining five race categories, χ^2^_(8)_ = 394.61, *p* < 0.001. Specifically, as shown in Fig. 3b, the proportion of Black arrestees with UDS results positive for FEN (not MET) was significantly larger than expected while the proportions of White and Asian arrestees with UDS results positive for FEN (not MET) were significantly smaller than expected. The proportion of White arrestees with UDS results positive for MET (not FEN) was also significantly smaller than expected, whereas the proportions of Black and Asian arrestees with this UDS result was significantly larger than expected under the null of no association between UDS result and race. The proportion of Black arrestees testing positive for FEN and MET was significantly smaller than expected, and the proportion of White arrestees testing positive for FEN and MET was significantly larger.

Lastly, age was significantly associated with UDS results positive for FEN and/or MET in the overall sample, *χ*^2^_(3)_ = 852.06,* p* < 0.001. Specifically, the proportions of adolescent (*z* = −16.94), young adult (*z* = −3.07), and senior (*z* = −4.76) arrestees positive for this group were significantly smaller than expected, while the proportion of UDS results positive for FEN and/or MET was significantly larger than expected for adult arrestees (*z* = 12.46). Figure 3c depicts the significant proportional differences observed when UDS results across age were examined for the three UDS positive subgroups, χ^2^_(6)_ = 415.03, *p* < 0.001. Proportions of UDS results positive for FEN (not MET) were significantly larger than expected among adolescent, young adult, and senior arrestees and significantly smaller than expected for adult arrestees. Significantly smaller proportions of MET (not FEN) were seen among adolescent and young adult arrestees, whereas adult and senior arrestees evidenced significantly larger proportions than expected. The proportion of adolescents with UDS results positive for FEN and MET were not significantly different from what was expected. Conversely, the proportion of young adults with UDS results positive for FEN and MET was significantly larger than expected, while the proportions of adult and senior arrestees with UDS results positive for FEN and MET were significantly smaller than expected.

### Fentanyl and/or methamphetamine (FEN and/or MET) subgroups plus other drugs

**Fig. 4:**
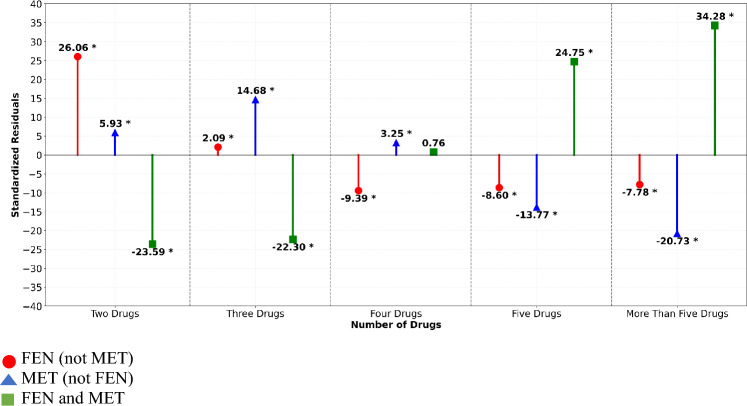
Standardized residuals for UDS cases positive for total number drugs by fentanyl and/or methamphetamine (FEN and/or MET) subgroups. Chi-square results reflect tests of association between for a given FEN and/or MET subgroup and specified number of total drugs category. *Difference in proportions significant at p < 0.05 based on standardized residual

**Fig. 5:**
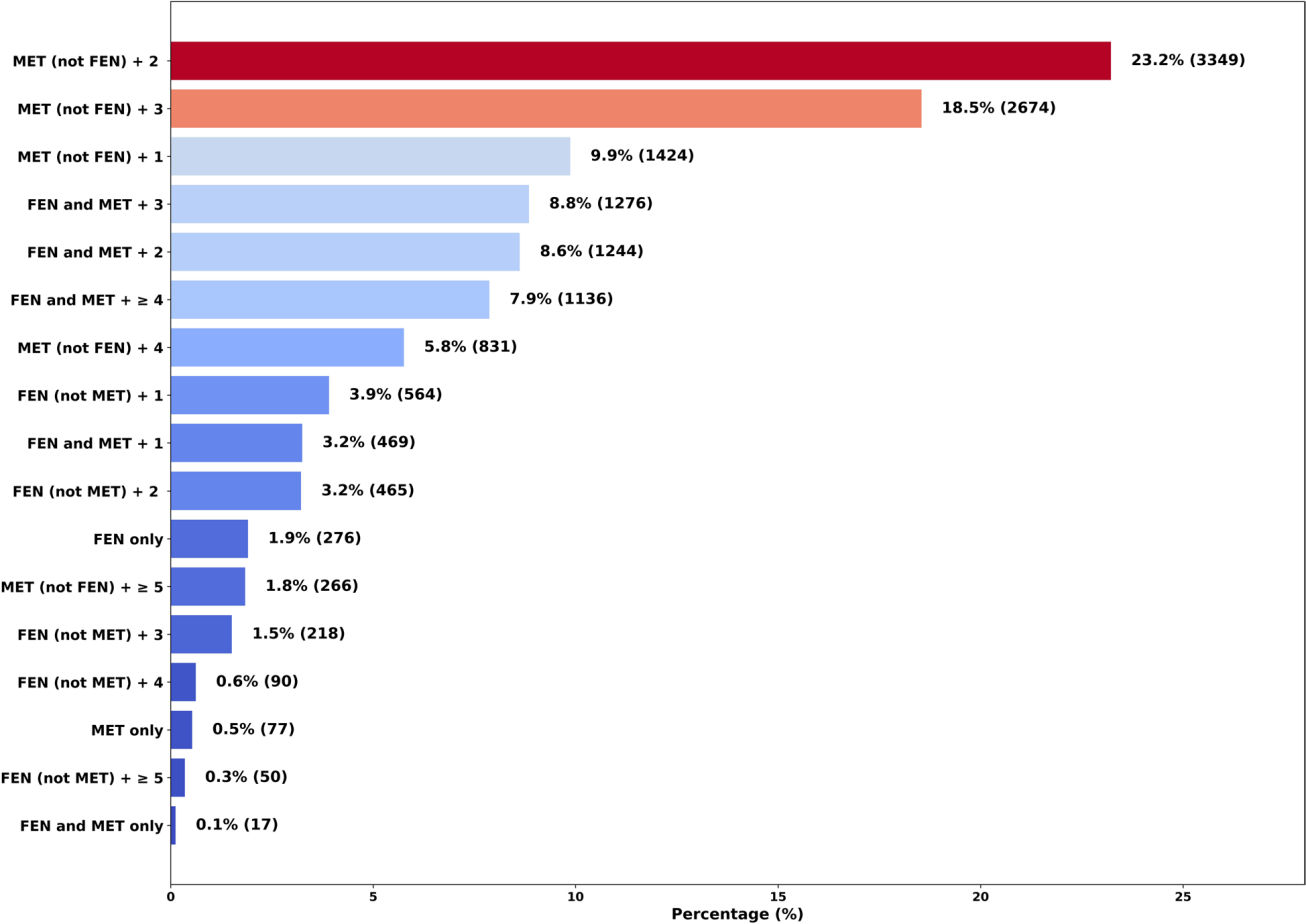
Frequencies and percentages of fentanyl and/or methamphetamine (FEN and/or MET) subgroups plus other drugs from most common to least common. Variable definitions for FEN and/or MET variables are found in the Methods section

Table 3 shows frequencies and percentages of UDS cases positive for FEN and/or MET subgroups plus other drugs by number of total drugs rank ordered from most to least common. This table shows the small percentage of arrestees (2.5%) who were UDS positive for FEN (not MET) and MET (not FEN) only, that is, with no other drugs. Among first-time arrestees with positive UDS for two or more drugs (*n* = 14,073, 97.5% of study sample), the subgroups were significantly associated with the number of drugs indicated by UDS testing, *χ*^2^_(8)_ = 4628.98,* p* < 0.001. Figure 4 shows the proportion of FEN (not MET) results was significantly greater than expected among individuals testing positive for two (*z* = 26.06) and three (*z* = 2.09) drugs, but smaller than expected among individuals testing positive for four (*z* = −9.39), five (*z* = −8.60), and more than five (*z* = −7.78) drugs. The proportion of MET (not FEN) was also significantly greater than expected among individuals testing positive for two (*z* = 5.93) and three (*z* = 14.68) drugs, as well as among those with UDS results positive for four drugs (*z* = 3.25); however, the proportion of MET (not FEN) was significantly smaller than expected among individuals testing positive for five (*z* = −13.77) or more than five (*z* = −20.73) drugs. Conversely, a significantly smaller proportion of individuals with UDS results positive for FEN and MET plus one or more other drugs—or just for both FEN and MET in the case of the “two drugs” category—was observed among those testing positive for two (*z* = −23.59) and three (*z* = −26.00) drugs and significantly larger than expected among individuals testing positive for five (*z* = 24.75) or more than five (*z* = 34.28) drugs. The proportion of individuals with UDS results positive for four drugs who tested positive for FEN and MET plus two other drugs was consistent with the frequency expected under the null. Taken together, these results seemingly suggest that individuals with positive UDS results for five or more drugs are notably more likely to test positive for both FEN and MET, whereas individuals with UDS results positive for four or fewer drugs were less likely to be positive for combined FEN and MET. FEN and/or MET subgroups plus other drugs are illustrated in Fig. 5 from most common to least common in the study sample. Positive UDS involving MET (not FEN) was most prominent, with MET (not FEN) + 2, MET (not FEN) + 3, and MET (not FEN) + 1 representing the top three patterns and accounting for approximately 51.6% of the study sample.

**Table 3:** Frequencies and percentages of UDS cases positive for fentanyl and/or methamphetamine (FEN and/or MET) subgroups plus other drugs by number of total drugs rank ordered from most to least common

Number of total drugs	Rank order		
	1st	2nd	3rd
One drug *n* = 353 (2.5%)	FEN (not MET) + 0	MET (not FEN) + 0	
*n*	276	77	
%	78.2	21.8	
Two drugs *n* = 2005 (13.9%)	MET (not FEN) + 1	FEN (not MET) + 1	FEN and MET + 0
*n*	1424	564	17
%	71.0	28.1	0.8
Three drugs *n* = 4283 (29.7%)	MET (not FEN) + 2	FEN and MET + 1	FEN (not MET) + 2
*n*	3349	469	465
%	78.2	10.9	10.86
Four drugs *n* = 4136 (28.7%)	MET (not FEN) + 3	FEN and MET + 2	FEN (not MET) + 3
*n*	2674	1244	218
%	64.6	30.1	5.3
Five drugs *n* = 2197 (15.2%)	FEN and MET + 3	MET (not FEN) + 4	FEN (not MET) + 4
*n*	1276	831	90
%	58.1	37.8	4.1
More than 5 drugs *n* = 1452 (10.0%)	FEN and MET + (≥ 4)	MET (not FEN) + (≥ 5)	FEN (not MET) + (≥ 5)
*n*	1136	266	50
%	78.2	18.3	3.4

Variable definitions for FEN and/or MET subgroups are found in the Methods section

## Discussion

This study builds upon prior research on UDS positivity among first-time arrestees across 25 U.S. jails [22] by focusing specifically on fentanyl, methamphetamine, and their combined presence among incarcerated individuals. Given that 44.3% of arrestees who tested positive for any drug were positive for fentanyl and/or methamphetamine, this study highlights the notable prevalence of these substances in correctional settings. While some of this prevalence may be attributed to facility policies on voluntary UDS testing, it also likely reflects the increasing arrests related to fentanyl and methamphetamine and their increased use in general [19, 20].

Among those in the study sample testing positive for fentanyl and/or methamphetamine, 59.8% were positive for methamphetamine without fentanyl, 28.7% for both fentanyl and methamphetamine, and 11.5% for fentanyl without methamphetamine. These findings align with national trends showing a rise in methamphetamine use disorder and overdose cases, as well as with studies showing an increased likelihood of fentanyl contamination in methamphetamine [12, 23]. The implications for correctional healthcare systems are significant and warrant consideration of expanded naloxone availability to address potential fentanyl overdoses, as some testing kits may not accurately detect fentanyl [11, 23].

Demographically, fentanyl and/or methamphetamine positivity was highest among White, male adults aged 40–59 years, consistent with national prisoner data from the U.S. Department of Justice (DOJ) [1]. However, DOJ reports also indicate a higher prevalence of methamphetamine use among incarcerated women compared to men. Individuals testing positive for both fentanyl and methamphetamine (i.e., co-occurrence) were predominantly white, young adults (aged 20–39), with similar co-occurrence rates in males and females. Geographic comparisons revealed fentanyl positivity was most common in Southern and Midwestern jails and methamphetamine positivity most common in Southern jails, whereas the odds of their co-occurrence were proportionally highest in Western jails. Jail size also played a role, with fentanyl-only cases more prevalent in mega jails, while combined fentanyl and methamphetamine were more common in medium-large and large jails. These findings emphasize the urgent need for medications for opioid use disorder (MOUD) in jails nationwide. Although fewer than half of U.S. jails offer MOUD, among all incarcerated individuals with opioid use disorder, less than 15% receive it [7]. Expanding MOUD access and training jail staff to recognize and address substance use disorders are potential priority pathways for combatting Fourth wave perils.

Although methamphetamine was notably more common among first-time arrestees testing positive for multiple drugs compared to fentanyl, polysubstance use patterns revealed fentanyl was still commonly detected when two or more drugs were present. Analysis also demonstrated that the combined presence of fentanyl and methamphetamine increased with higher levels of polysubstance use. This finding mirrors trends observed in prior studies, where 13.2% of justice-involved individuals in Kentucky jails reported daily use of both opioids and stimulants (Bunting et al. 25), and in San Diego where 97% of individuals who tested positive for multiple substances were positive for methamphetamine, while 21% tested positive for opiates [21]. Results from Schumacher et al. [22] further supported this trend, showing 48.9% of first-time arrestees tested positive for multiple substances, with 53.0% testing positive for cannabis and stimulants, and 25.1% for opioids and stimulants.

Taken together, these findings suggest fentanyl and methamphetamine use rarely occur in isolation; rather, their presence increases in the context of broader polysubstance use. As the number of drugs detected in UDS increases, the likelihood of a fentanyl-methamphetamine co-occurrence rises, underscoring the complexity of UDS patterns observed in correctional settings and hinting at the intricacy of drug use trends in incarcerated populations.

### Limitations

This study is not without limitations that must be considered when interpreting findings. First, the clinical and demographic data collected were primarily for administrative purposes, which may limit their comprehensiveness for epidemiologic analysis. Additionally, because UDS testing was largely based on self-reported substance use history, there is a strong likelihood of underreporting, particularly among individuals whose arrests were drug-related. Future research should utilize the few sites that administer UDS tests to all individuals and compare rates of underreporting to strengthen understanding of this limitation. Another methodological limitation is that drug identification relied solely on UDS without confirmatory testing (e.g., GC–MS or LC–MS), which introduces the possibility of false positives and may overestimate actual drug exposure. Confirmatory testing data were not available; future studies should include a prospective, random sample of arrestees undergoing both UDS and confirmatory testing to assess false positive rates and improve prevalence accuracy. Another limitation is the inability to distinguish between intentional and unintentional fentanyl or methamphetamine use, as increasing reports are suggesting individuals may unknowingly consume fentanyl-laced methamphetamine [23]. This inability complicates interpretation of prevalence estimates, as it is unclear whether positive fentanyl results reflect intentional use, contamination, or an unrecognized co-use pattern. With regard to testing policy, all participating jails applied the same criterion, UDS testing based on suspected withdrawal risk, except for one site that used universal testing and was excluded from analysis. Compliance rates could not be calculated due to the absence of data indicating how many arrestees were eligible but declined testing,future research could examine screening data to determine who should have received UDS based on risk status. Furthermore, voluntary UDS testing introduces potential selection biases, as systematic differences might exist in the types of arrestees perceived by staff to warrant UDS and arrestees who declined testing might meaningfully differ from those who were ultimately administered a UDS (e.g., potentially different substance use patterns). Such selection biases potentially led to an underestimation of the true prevalence of fentanyl and methamphetamine use among first-time arrestees and might have introduced or intensified group differences in positivity rates. Despite these limitations, the present study provides valuable insights into UDS result patterns, possibly generalizing to drug use patterns, among arrestees. The results of this study also have implications for future research and practice, as findings reflect the data available to correctional and healthcare staff when making decisions regarding intervention and policy implementation.

### Implications for future research & practice

Above all else, the findings of this study highlight the urgent need for continued and improved screening, treatment, and intervention strategies for substance use in jail settings. Given the high prevalence of fentanyl and methamphetamine-positive UDS results, expanding standardized screening protocols and increasing the availability of medications for opioid use disorder (MOUD) in correctional facilities warrants priority attention. Given the high rate of positive UDS results among first-time arrestees, jail-based interventions present a critical opportunity to alter the trajectory of substance use early in the criminal justice process. Additionally, training jail staff and healthcare professionals to recognize signs/symptoms of substance use, withdrawal, and overdose can enhance early intervention efforts and improve overall care for incarcerated individuals. Future research should focus on assessing the prevalence of unintentional fentanyl exposure among methamphetamine users to better inform harm reduction strategies. Furthermore, longitudinal studies tracking substance use and overdose risk post-release and across bookings would provide critical data on the effectiveness or shortfalls of jail-based interventions and highlight the need for enhanced continuity of care. Addressing these issues through policy changes, education, and expanded treatment accessibility can help mitigate the risks associated with polysubstance use among correctional populations and contribute to broader public health improvements.

## Conclusion

This study highlights the high prevalence of fentanyl and/or methamphetamine use among first-time arrestees, reinforcing the need for targeted interventions, expanded in-jail substance use treatment, and comprehensive post-release support. The intersection of polysubstance use, demographic trends, and regional variations underscores the importance of MOUD accessibility, staff training, and improved screening measures in correctional facilities. Successful implementation of MOUD programs in jails, such as those in Rhode Island and select counties in New York and California, has demonstrated reductions in post-release overdose deaths and improved engagement in community-based care [10, 18]. Given the growing complexities of the opioid and stimulant crisis, integrating evidence-based harm reduction strategies within jails and ensuring continuity of care post-release will be critical to reducing overdose deaths and improving public health outcomes.

## Data Availability

Data were de-identified and provided to the authors under a confidential data sharing agreement between NaphCare, Inc. (NaphCare) and NaphCare Charitable Foundation, Inc. (NCF) and so are not publicly available.
